# Terrestrial isopods in urban environments: an overview

**DOI:** 10.3897/zookeys.801.29580

**Published:** 2018-12-03

**Authors:** Katalin Szlavecz, Ferenc Vilisics, Zsolt Tóth, Elisabeth Hornung

**Affiliations:** 1 Department of Earth and Planetary Sciences, The Johns Hopkins University, 3400 N. Charles St., Baltimore, MD 21218-2681, USA The Johns Hopkins University Baltimore United States of America; 2 Freelancer biologist, CEO of Makkaramies Oy, Helsinki, Finland Unaffiliated Helsinki Finland; 3 Department of Ecology, Institute for Biology, University of Veterinary Medicine Budapest, H-1077 Budapest, Rottenbiller str. 50., Hungary University of Veterinary Medicine Budapest Budapest Hungary

**Keywords:** Adaptation, anthropogenic habitats, biotic homogenization, ecosystem services, soil fauna

## Abstract

In an increasingly urbanized world scientific research has shifted towards the understanding of cities as unique ecosystems. Urban land use change results in rapid and drastic changes in physical and biological properties, including that of biodiversity and community composition. Soil biodiversity research often lags behind the more charismatic groups such as vertebrates and plants. This paper attempts to fill this gap and provides an overview on urban isopod research. First, a brief overview on urban land use change is given, specifically on the major alterations on surface soils. Historical studies on urban isopods is summarized, followed by the status of current knowledge on diversity, distribution, and function of urban isopod species and communities. A review of more than 100 publications revealed that worldwide 50 cities and towns have some record of terrestrial isopod species, but only a few of those are city-scale explorations of urban fauna. A total of 110 isopod species has been recorded although the majority of them only once. The ten most frequently occurring isopods are widely distributed synanthropic species. Knowledge gaps and future research needs call for a better global dataset, long term monitoring of urban populations, multi-scale analyses of landscape properties as potential drivers of isopod diversity, and molecular studies to detect evolutionary changes.

## Introduction

In 2008 humans reached a major milestone: more than 50% of the global population now lives in cities (United Nation Population Division, World Urbanization Prospects 2011). Urbanization has become the major type of land use change in the 21^st^ century. Conversion of wild lands or of former agricultural land to urban, suburban and exurban areas fundamentally alter the landscape and its biodiversity. Changes in the soil subsystem are especially dramatic. During construction, physical clearing, removal of upper horizons, filling, and mixing result in the loss of natural soil profiles. Urban soils are often highly compacted, have low hydraulic conductivity and low organic matter content. Soil sealing is one of the most visible surficial changes. Impervious surfaces disrupt the connection between the soil and the atmosphere, altering runoff, infiltration, nutrient input and gas exchange. It restricts or entirely prevents movement of soil organisms between the surface and deeper soil. Additionally, roads, pavements and other built structures lead to an even more heterogeneous landscape, acting as barriers for horizontal movement. At the same time, underground pipe systems can serve as corridors connecting habitat fragments. Chemical alteration of soils is also significant and is related either to accidental events (e.g., leakage of ageing sewer systems, spillage of contaminants), or direct management (e.g., application of road salts, fertilizers, and pesticides) (Pouyat et al. 2007, 2010). Soil degradation and soil sustainability are of national and global concern as described in the recent report by the European Academies (van der Putten et al. 2018).

In the urban-suburban landscape the habitat unit is often the parcel, a piece of land owned by private citizens, organizations (e.g., neighborhood associations), companies, or other groups of landowners. Decisions about land management happen at this scale further increasing spatial heterogeneity. Individual homeowners or small groups decide on planting, mulching, irrigation, and usage of fertilizers and pesticides. Large amounts of soil and other landscaping material are moved around, overcoming natural or man-made barriers. All these accidental or deliberate actions affect distribution and abundance of soil organisms including that of terrestrial isopods.

While urban land conversion often destroys existing habitats, it also creates new ones. Urban habitat patches range from remnants of the ’natural’ community through more or less disturbed and/or managed habitats (e.g., parks, backyards, industrial grounds) to entirely novel habitats, such as green roofs, greenhouses, or even soilless environments such as basements and underground pipe systems. This wide spectrum of habitat types may result in overall higher species richness than expected. On the one hand, remnant patches of the native vegetation can sustain populations of the regional soil fauna. On the other hand, the physical environment in the novel habitats often allow the existence of species that otherwise would not survive under the normal climatic conditions. Cities are often viewed as hot-spots for non-native species introduction due to high traffic and trade (Vilisics and Hornung 2009).

Major restructuring of the surface-subsurface affects not only biogeochemical processes, but the biota mediating these processes as well. Soil fauna and microorganisms are key players in regulating pathways and rates of decomposition, thereby affecting storage and release of carbon, nitrogen and other nutrients. Endogeic fauna can modify porosity, which affects water holding capacity, infiltration, and gas diffusivity. Thus, urban soils provide the same ecosystem services that naturally developing or agricultural soils do (Millennium Ecosystem Assessment 2005, Pavao-Zuckerman 2013).

The relative importance of natural vs. anthropogenic drivers in structuring urban communities and controlling ecosystem functions is variable, but generally the latter is viewed as a dominant force (Alberti 1999, Kaye et al. 2006). According to the Urban Ecosystem Convergence Hypothesis (Pouyat et al. 2003), this leads to more similar soil characteristics across regional or global scales compared to the soils they replaced. Data on soil organic matter and pH from five cities has supported this hypothesis (Pouyat et al. 2015a). Urbanization also results in more similar soil fauna, a process known as Biotic Homogenization (McKinney 2006, Olden et al. 2016). Two major components of biotic homogenization in urban areas are the extinction of local fauna, and the transportation of non-native, usually synanthropic species across geographical boundaries.

In this review we summarize past and current research on terrestrial isopods in urban environments. First, we provide an overview on the history of urban isopod research. We then summarize major findings, and highlight research gaps. For species names we follow the nomenclature by Schmalfuss (2003).

## Research on urban isopods – history

Early studies on urban isopods were mostly zoological surveys in the neighboring parks, backyards or as part of regional fauna assessments (e.g., Flasarová 1995). Specialized habitats such as botanical gardens and greenhouses have always been favored by zoologists; papers with species records go back to the turn of the 20^th^ century (e.g., Bagnall 1909, Holthuis 1945, for full list see Schmalfuss 2002). Greenhouses and botanical gardens with many exotic plants and introduced soil were promising habitats to discover species new to the region or even to science (Korsós et al. 2002, Kontschán 2004, De Smedt et al. 2017). Exploration of these green spaces still continues (Montesanto 2015, De Smedt et al. 2017). The early records of species occurrences are extremely valuable, as we can use this historical information to document changes in the local fauna. For instance Montesanto (2015) reexamined isopod fauna from the botanical garden in Pisa, Italy. While total number of species did not change much (seven species between 1914–16, and eight species in 2014–15), species composition did: only one species, *Armadillidiumdepressum* Brandt, 1833, was recorded in both time periods.

Ecological studies on urban isopods coincide with the rise of urban ecology as a discipline in the 1980–90s (e.g., Klausnitzer 1987, Trepl 1995, Sukopp 1998), and were predominantly conducted in Europe (e.g., Tischler 1980, Kühnelt 1989, Schulte et al. 1989). In the US, the establishment of two urban Long Term Ecological Research (LTER) sites, Phoenix AZ, and Baltimore MD, launched urban ecosystem research in 1997. Information on isopod species composition and abundance is often published as part of a broader study specifically focusing on urban soil fauna (e.g., Schaefer 1982, Fründ et al. 1989, Smith et al. 2006a, Jordan and Jones 2007, Bolger et al. 2000). Finally, a body of literature exists on the effects of pollutants, especially heavy metals on isopods. These organisms are known to accumulate heavy metals, and thus have been used as indicators of pollution levels in cities (Dallinger et al. 1992, Hopkin et al. 1993, Komarnicki 2005, Pedrini-Martha et al. 2012).

The development of better theoretical framework and methodological approaches (Grimm et al. 2000, Pickett et al. 2008) shifted urban ecology from descriptive to more mechanistic (Shochat et al. 2006). Attempts to explain biodiversity in urban habitat patches include the theory of island biogeography (Fründ and Ruszkowsky 1989), the intermediate disturbance hypothesis (Niemelä et al. 2000), and more recently, the metacommunity theory (Swan et al. 2011). One influential concept in early urban ecosystem research is the urban-rural gradient approach (McDonnell et al. 1993), which was adopted in various urban research programs including the GLOBENET Project (Niemelä et al. 2000), an international research initiative. In this project replicates of urban, suburban and rural forest patches were established to compare soil fauna and test the hypotheses about the connection between disturbance and diversity, and the effects of urbanization on morphological traits. Originally designed to study carabid beetles, the project extended to other epigeic arthropods, including isopods (Magura et al. 2008a, 2008b, Vilisics et al. 2007a, Hornung et al. 2007b).

**Figure 1.**
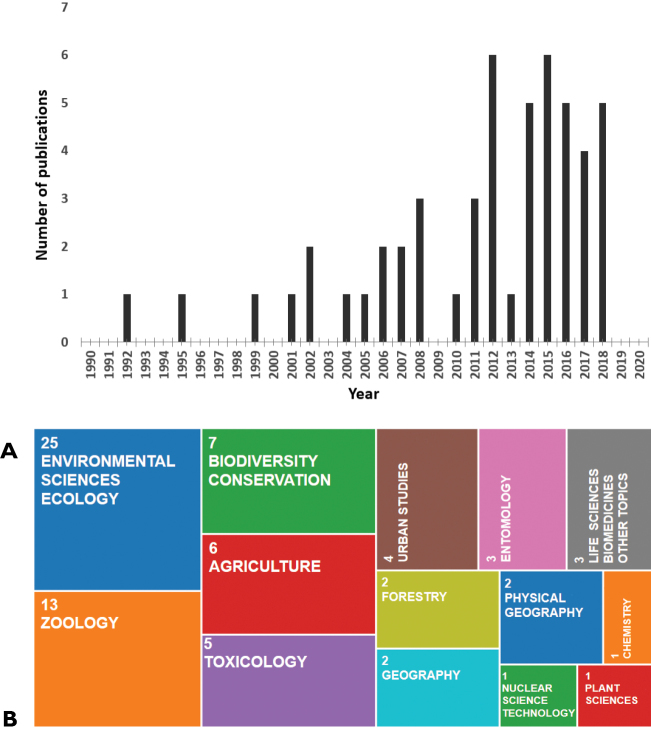
Publications on urban isopods in the past three decades. Source: Web of Science using the following keywords: terrestrial isopods, woodlice, oniscid, urban, anthropogenic **A** Number of publications per year **B** Frequency of publications by subdisciplines.

**Figure 2.**
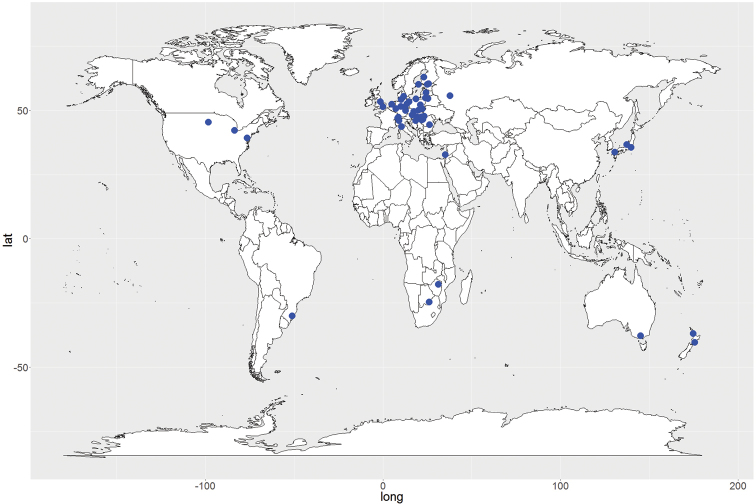
Cities with records on urban isopod diversity. Names of cities with references are listed in Suppl. material 1: Table S1. Publications with isopod abundance but without species composition are not included in the map, but referenced elsewhere in the text.

With the growing interest in cities as ecological systems, the number of publications focusing in urban soil fauna grew, and isopod literature followed this trend (Figure 1A). More and more cities have conducted isopod surveys although the global distribution of these studies is heavily biased towards European cities (Fig. 2). The research topics further diversified (Figure 1B) and today include observations on altered behavior of isopods in urban environments (Houghtaling and Kight 2006, Cividini and Montesanto 2018a, 2018b). To date a total of 50 cities have some records of isopod species (Suppl. material 1: Table S1). These cities vary in size, age, geographical location, major landscape features (e.g., types and coverage of green spaces), and land use type of the surrounding matrix.

## Terrestrial isopods in cities

### Diversity and distribution

In any urban area species richness consists of two components: a subset of the regional pool of native species, and a group of accidentally or intentionally introduced species. Oniscidea form a diverse group with regard to size, mobility (thus dispersal ability), and tolerance to environmental extremes (Schmalfuss 1984, Warburg 1993, Hornung 2011). Therefore, the highly fragmented urban landscape will affect local fauna (and thus alpha diversity) differently. In general, local endemic species may disappear, and can be replaced by synanthropic, widely distributed species. Native habitat specialists (e.g., species requiring coarse woody debris, bogs or ravines) are especially vulnerable and thus are often extirpated. However, this is not always the case: even local endemics can survive in small pockets of natural remnants within the city matrix (Vilisics and Hornung 2009) similarly to isopods in forest fragments embedded in an agricultural landscape (Tajovský et al. 2012). Unmanaged gardens and yards can also serve as refuges for native species (Hornung et al. 2007, Vilisics and Hornung 2009, Vilisics et al. 2012, Hornung et al. 2018).

Local microhabitat characteristics are one major determinant in the survival of species. For instance, ruins of medieval castles or other historical buildings, stone walls or large slabs of concrete provide suitable shelter for *Porcelliospinicornis* Say, 1818 even in close proximity to the city core (Vilisics 2007). In general, neglected areas that often lack tall vegetation, but are abundant in hard surfaces, abandoned brownfield and construction sites and crumbling medieval ruins share similar site characteristics for isopods. These areas often support less common, drought tolerant species (e.g., *Porcelliodilatatus* Brandt, 1833, *Porcelliospinicornis* and *Porcellionidespruinosus* (Brandt, 1833)) (Vilisics and Hornung 2009, 2011). Native species may appear remarkably resistant to disturbances brought about by urbanization and successfully colonize many urban habitats. Examples are *Porcelliumcollicola* (Verhoeff, 1907) in Europe, and *Balloniscusglaber* Araujo & Zardo, 1995 in Brazil. The latter species has been found at high abundances in the city of Porto Alegre, RS, despite being categorized as a habitat specialist and K-strategist (Kenne and Araujo 2015). These species can be examples of ’urban adapters’ a term coined by Blair (1996) and McKinney (2006), although it is unclear if adaptation in the evolutionary sense has occurred.

The review of urban isopod records revealed a list of over 100 species belonging to 15 families (Suppl. material 2: Table S2). The majority of these species (70 in total) has been recorded only once. *Porcellioscaber* Latreille, 1804, *Trachelipusrathkii* (Brandt, 1833) and *Armadillidiumvulgare* Latreille, 1804, occur in more than 50% of the cities listed in Suppl. material 1: Table S1, with the latter being the most frequent. The other, frequently occurring species are also well known, widely distributed synanthropic species, including *Oniscusasellus* Linnaeus, 1758, *Philosciamuscorum* (Scopoli, 1763), *Porcellioscaber, Porcellionidespruinosus, Hyloniscusriparius* (C. Koch, 1838) and *Cylisticusconvexus* (De Geer, 1778) (Table 1). These species can also be common in more ’natural’ areas far from the urban core. For instance, *Trachelipusrathkii* can dominate on floodplains (Farkas 1998, Tajovský 1998), and *Armadillidiumvulgare* is common on sandy shores (Hassall and Sutton 1978) and in forest remnants of the Hungarian Great Plains (Szlavecz 1991). The repeated presence of these species leads to greater similarity among urban isopod faunas. However, we need to emphasize that urban species records are often incomplete. Surveys are often restricted for a particular habitat type (parks, remnant forest patches, riparian areas or vacant lots), and/or are short term studies. Large scale surveys are needed to explore how many of these common, synanthropic species have been established in climatic regions and biomes outside of their more natural range, and how many rare species persist in less studied, unique habitats.

**Table 1.** List of the ten most common Isopoda in urban environments. References not older than 70 years were used for this list. Full list of species is in Suppl. material 2: Table S2, and references are listed in Suppl. material 1: Table S1.

Species	Family	Percentage of records (N = 50)
* Armadillidium nasatum *	Armadillidiidae	38
* Armadillidium vulgare *	Armadillidiidae	72
* Cylisticus convexus *	Cylisticidae	44
* Haplophthalmus danicus *	Trichoniscidae	38
* Hyloniscus riparius *	Trichoniscidae	42
* Oniscus asellus *	Oniscidae	42
* Porcellionides pruinosus *	Porcellionidae	48
* Porcellio scaber *	Porcellionidae	68
* Porcellio spinicornis *	Porcellionidae	38
* Trachelipus rathkii *	Trachelipodidae	56

What fraction of the regional species pool survives in a city depends on the type and strength of environmental filtering, and tolerance and adaptability of the native species. Both are related to geological and land use history of the region. Cities can harbor a large fraction of the regional native Oniscidea fauna. In Budapest, Hungary, Korsós et al. (2002) recorded 38% of the then known isopod fauna of Hungary; since then several new species have been added to the fauna list (Vilisics 2007) elevating this number. In Warsaw, Poland, 60% of the regional fauna was recovered in urban-suburban areas (Jedryczkowski 1981). Out of the 30 species recorded in rural habitats in NE Bohemia, Czech Republic, 17 occurred also in urban parks and gardens (Flasarová 1995). Similar total species richness numbers were reported from Bucharest, Romania (Giurginca 2006), Kiel, Germany (Tischler 1980), Leipzig, Germany (Arndt and Mattern 1996), and Olomutz, Czech Republic (Riedel et al. 2009). Broken down to main habitat types, gardens have been shown to support high diversity in Budapest, Hungary (Vilisics and Hornung 2009), Warsaw, Poland (Jedryczkowski 1981) and London, England (Smith et al. 2006b). At the other end of the spectrum are regions with low or zero native species richness. In North America, although approximately two thirds of the 115 listed species are endemic, most native species are restricted to the southern states, coastal areas and caves (Jass and Klausmeier 2000). Inland isopod faunas in the Atlantic, Midwest, and in Canada consist almost entirely of exotic, cosmopolitan, synanthropic isopods (Leistikow and Wägele 1999, Jass and Klausmeier 2000). Most likely they were introduced from Europe when ships carried soil as ballast material and in plant containers brought along by immigrants (Lindroth 1957). Lack of competition with native counterparts likely enhanced successful colonization and spread of these species. They became widespread and abundant in wildland areas, such as forests, grasslands, or wetlands, as well as in agricultural fields and cities (e.g., Hatchett 1947, Sorensen and Burkett 1977, Hornung et al. 2015). In this situation the regional and local species composition overlap (Hornung and Szlavecz 2003). Reconstructing the origin and spread of these non-native species is challenging, but molecular techniques greatly enhanced our ability to reveal the past (Lee et al. 2014). For instance, combining historical records with molecular studies on European and North American populations of *Armadillidiumvulgare*, Garthwaite et al. (1995) suggested that the species first was introduced to greenhouses, and later spread throughout the Unites States. Molecular studies also revealed that the East Coast and West Coast were independently colonized by founder populations from different latitudes in Europe, which corresponds to the known history of European settlement in North America.

Many exotic species do not establish successfully in the new environment, although this is difficult to demonstrate. Others sustain populations on the long-term, but are still restricted to man-made environments. *Trichorhinatomentosa* (Budde-Lund, 1893), *Buddelundiellacataractae* Verhoeff, 1930, and *Armadillidiumnasatum* Budde-Lund, 1885 are common in greenhouses across Europe, although the latter is somewhat different. *Armadillidiumnasatum*, often called ‘greenhouse pillbug’, has adapted to outdoor conditions and occurs in many areas north of its native range (Allspach 1987, Berg et al. 2008, Vilisics et al. 2012). It is also common in the Mid-Atlantic region in North America, where it was found in suburban gardens, parks, forests, and cropfields (Hornung and Szlavecz 2003, Szlavecz unpubl.). Interestingly, even populations of synanthropic species can decline over time. Harding (2016) speculated that the recent decrease in *Porcelliolaevis* (Latreille, 1804) localities in Britain might be associated with the decrease of suitable synanthropic sites.

Many cities have been built in regions where the climatic conditions are outside of the tolerance limit of most isopods. These settlements may still harbor high abundance of organisms because the way humans modify the landscape and microclimate, shifts habitat conditions towards more optimal. In metropolitan Phoenix, Arizona, where the natural biome is desert, changes in vegetation cover and regular irrigation shifted residential yards from xeric to mesic. The abundance of isopods increased by a factor of 50, and they became the second most abundant macroarthropod group following ants (Cook and Faeth 2006). At high latitudes, where persistent cold temperatures limit isopod distribution in wildlands (e.g., Kuznetsova and Gongalsky 2012), urban areas provide favorable microhabitats. In Finland, isopods were found in urban forests and parks but not in rural forests (Vilisics and Terhiuvo 2009). Urban woodlands have more broadleaf tree species providing food and shelter for these detritivores. Heated buildings have been shown to be essential for winter survival of several mesic isopod populations in South Dakota, USA (Wright 1997). The isopods have only modest cold tolerance and suffer high mortality in the winter unless they migrate to great depth, or overwinter in the cracks and crevices of heated houses. In such extreme cold climates isopods exhibit extreme synanthropy. Future climate and land use change may open up new areas for colonization from these urban centers expanding the boundaries of distribution.

### Abundance of isopods in urban environments

Regardless of diversity, urban habitats support large abundances of isopods. Isopods have been shown one of the most numerous group compared to other epigeic arthropods (Bolger et al 2000, Cook and Faeth 2006, Smith et al. 2006a, Vilisics et al. 2007a, Philpott et al. 2014) although opposite examples also exist (Norton et al. 2014) (Table 2). Higher abundance of isopods has been reported from urban parks and forests than in rural habitat types (Tischler 1980, Walton et al. 2006, Hornung et al. 2015).

**Figure 3.**
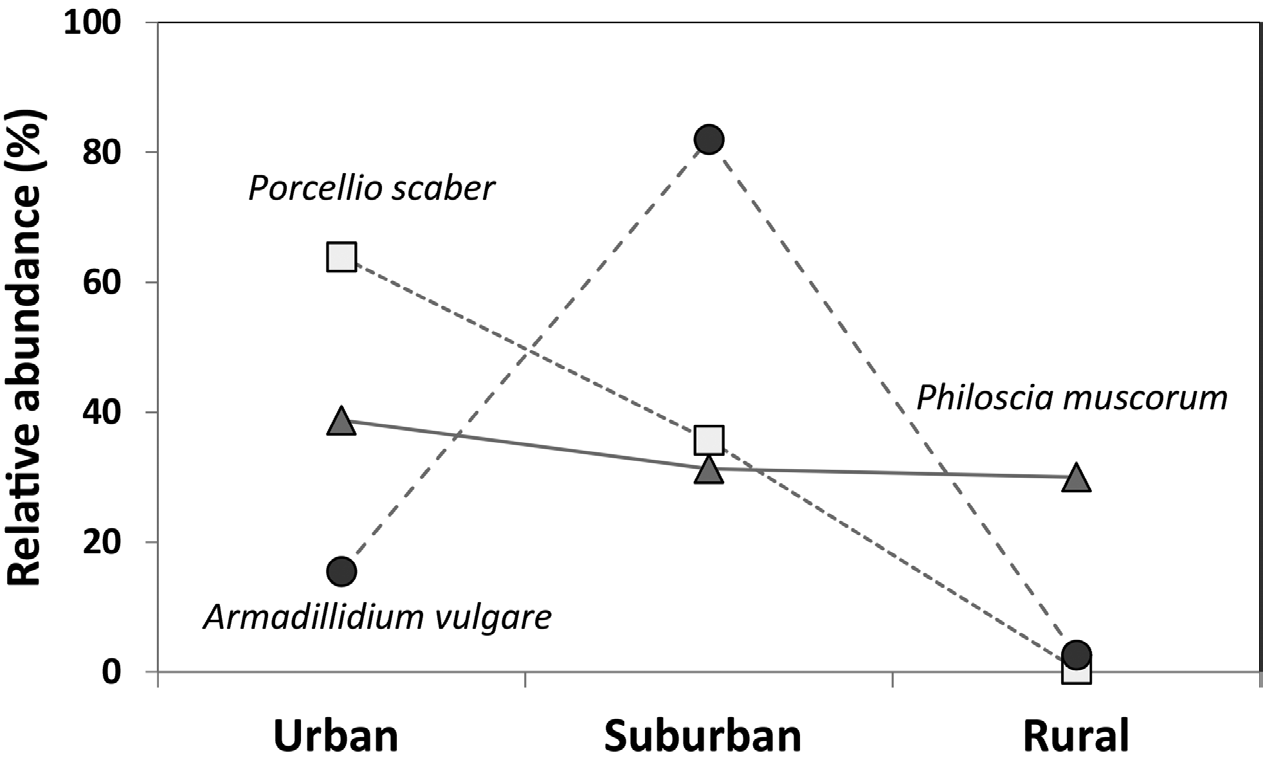
Responses of three synanthropic isopod species to urbanization gradient. Each data point is percentage of total number of individuals (N) of a given species caught in pitfall traps. *Philosciamuscorum*: N = 7473, *Porcellioscaber*: N = 12314, *Armadillidiumvulgare*: N = 816. The study was carried out in urban, suburban, and rural forest patches and parks in Sorø, Denmark. Data from Vilisics et al. (2007); original figure.

In the above mentioned GLOBENET project, pitfall trap material was analyzed in two cities: Sorø, Denmark, and Debrecen, Hungary. Isopod abundance was consistently higher in the urban core that in the rural habitats (Hornung et al. 2007, Magura et al. 2008a). Due to low overall species richness (5–6) there was no pattern regarding species composition. However, the relative abundance of species was different along the urban-rural gradient (Figure 3), indicating a species level response to urbanization. In Sorø, abundance of *Porcellioscaber* was an order of magnitude higher in urban parks than in rural forests, *Armadillidiumvulgare* and *Ligidiumhypnorum* (Cuvier, 1792) reached their maxima in suburban patches, and *Philosciamuscorum* was evenly distributed along the gradient (Vilisics et al. 2007a). Similar, species-specific response was detected in Debrecen, Hungary, as well (Magura et al. 2008a). It is important to note that pitfall trap sampling is selective, and small, slow moving, rare species, or species occupying special microhabitats (e.g., endogean species), can be missed.

**Table 2.** Relative abundance of terrestrial isopods in the urban and suburban landscape. Numbers are expressed as percentage of isopods in pitfall trap materials, thus reflecting their abundance in relation to epigeic arthropods.

Location†	Land use/cover type	Percentage of isopods	Reference
San Diego CA, USA	Various suburban	48	Bolger et al. 2000
Toledo OH, USA	Various	59	Philpott et al. 2014
Sheffield, UK	Gardens	45	Smith et al. 2006
Yorkshire, UK	Urban agriculture	51	Turnbull 2012
Baltimore MD, USA	Vacant lots	52	Szlavecz, unpubl.
Chicago IL, USA	Woodland fragments	34/55‡	McCary et al. 2017
Melbourne, Australia	Grass with variable cover	<1	Norton et al. 2014
Osaka, Japan	Variable	88	Lee and Kwon 2015
Phoenix, AZ, USA	Variable, mesic-xeric	1.9/12‡	Cook and Faeth 2006
Phoenix, AZ, USA	Irrigated residential yards	7/37‡	Cook and Faeth 2006

† City might include greater metropolitan area ‡ First number was reported in publication and includes all arthropods. Second number is isopod percentage after removing Collembola and Acari. Removal of microarthropods allows more realistic comparison of macrofauna relative abundances.

### Isopods as environmental indicators

In the 1980–90s the main focus of urban research was contamination, especially heavy metal pollution (van Gestel 2012). Due to heavy industry near cities, emissions from vehicular traffic and other fossil fuel burning, and intensive use or accidental spills of organic compounds, the general view was that urban soils have high concentrations of lead (Pb), cadmium (Cd), copper (Cu), zinc (Zn) and other metals as well as organic contaminants. A wealth of papers has been published about how exposure to contaminated sites and food affect morphology (Godet et al. 2012, Mazzei et al. 2014), physiology (Farkas et al. 1996; Hornung et al. 1998a, Zidar et al. 1998) life history (Eckwert and Köhler 1997, Kammenga et al. 2001, Calhôa et al. 2012) and behavior (Drahokoupilová and Tuf 2012) of isopods. These organisms are known to accumulate heavy metals, often to a greater degree than other invertebrates (Heikens et al. 2001). The ability of isopods to respond to environmental stress has made them ideal test organisms to evaluate environmental quality (Drobne and Hopkin 1995, Drobne 1997, Hornung et al. 1998b, Cortet et al. 1999, Caseiro et al. 2000), and to this day isopods are standard organisms in laboratory ecotoxicology tests (van Gestel 2012). Isopods were proposed to use as indicators of pollution levels in cities (Dallinger et al. 1992, Komarnicki 2005) and even at global scale (Hopkin et al. 1993). Recently Pedrini-Martha et al. (2012) showed that isopods are good indicators of mercury (Hg) contamination, while Papp et al. (2018) reported significant differences in Ba and Cu concentration of *Armadillidiumvulgare* individuals along an urbanization gradient. Heavy metal concentration in isopod and other invertebrate tissue can be significantly affected by sampling and sample preparation protocols, which is important to keep in mind when making between-city comparisons (Zödl and Wittmann 2003). A growing number of studies focus on organic pollutants (e.g., van Brummelen et al. 1996, Loureiro et al. 2005) which is a particularly serious environmental problem in urban soils. Agodi et al. (2015) has shown *Armadilloofficinalis* Dumèril, 1816 to be a promising indicator species to benzene exposure including the effect of this carcinogenic air pollutant on mitochondrial DNA. Since isopods are food source of vertebrate and invertebrate predators, one threat is the transfer and potential accumulation of these contaminants at higher trophic levels. In a regional assessment of the Greater Washington-Baltimore Metropolitan area, Pouyat et al. (2015b) found a positive correlation between lead concentration of soil, isopod body, and blood of American robin (*Turdusmigratorius* Linnaeus, 1766) nestlings from the same residential yards.

Fluctuating asymmetry (FA) has been proposed as a method to assess environmental quality (Clarke 1993). Developmental perturbations, due to environmental stress or genetic causes, results in deviations from bilateral symmetry and can be used as an indicator of levels of contamination. So far studies on isopod populations produced contradictory results. Both higher (Peters et al. 2001) and lower (Godet et al. 2012) levels of FA were reported from contaminated sites compared to control sites. Papp et al. (2018) found no difference along an urban-rural gradient. Exploring a different type of environmental stress, Vilisics et al. (2005) reported higher asymmetry at a locality with dramatic changes in soil moisture conditions, than from a more stable habitat.

Isopod assemblages can also reflect the level of disturbance and/or ‘naturalness’ of an area. Simple species numbers or diversity indices do not inform us about community composition. In cities, where non-native, synanthropic, and/or common species mix with native and/or rare ones, species diversity can be high, yet from a conservation point of view, the quality of the habitat is poor (Hornung et al. 2007, 2008, 2009, Vilisics and Hornung 2009). The ’Terrestrial Isopod Naturalness Index’ (TINI) proposes a scoring system to characterize the isopod species. Adding species scores of a community and divided by species number (Σ TINI/S, where S is the number of species) gives Average Rarity Index (ARI), which then indicates local environmental quality. Basic components of the scoring system include whether the species is (1) introduced, (2) well established, (3) synanthropic, (4) disturbance tolerant and (5) member of the fauna in the region under study. Each component of the index receives a score with a maximum value of 20 (Hornung et al. 2008, 2009, Hornung et al. 2018).

## Terrestrial isopods are a successful group in urban environments

The fact that isopods reach high population densities indicates that while species level differences clearly exists, as a group they can successfully colonize urban and suburban habitats. Multiple factors contribute to their success. First, as detritivores, they are food generalists capable of living on leaf litter, garden and kitchen refuse, and even on pet food. In some countries, it is common landscaping practice to cover tree bases, planting beds and bare soil surfaces with a thick layer of mulch. Mulch is often shredded woody material that retains moisture and, by slowly decomposing, adds organic matter to the soil surface (Byrne et al. 2008). This is both ideal sheltering habitat and food resource to isopods even in relatively open areas. Second, the built environment is rich in calcium (Ca) that isopods need. Calcium is present in the form of concrete dust, pavements, and landscaping limestone rocks, and gravel. Isopods are sensitive to soil acidity (van Straalen and Verhoef 1997), but land management can overcome this limitation: soil, too acidic for grass to grow, is amended with lime as part of the annual lawn care packages in the US. Third, another management practice, irrigation allows isopods to cope with their most significant abiotic limiting factors: low humidity and soil moisture, as demonstrated by the dramatic increase of isopod abundance in irrigated residential yards in a desert biome (Cook and Faeth 2006). Finally, the fourth component of their successful urban existence is that many species can live in soil-less substrates, primarily buildings. They can use the built environment, cellars, cisterns, and tool sheds temporarily in extreme weather conditions (Wright 1997), or permanently (e.g., Klausnitzer and Herr 1988). The Mediterranean species *Chaetophilosciacellaria* (Dollfus, 1884) and *Protracheoniscusmajor* (Dollfus, 1903) of Central-Asian origin are examples of domicole species in Central Europe (Vilisics and Hornung 2009). Isopods are moderate dispersers, but can be easily moved with plants, topsoil, compost, mulch, and even trash. Isopods are also used as pet food which further increases the chance of being transported to new areas. For instance, juvenile *Trichorhinatomentosa* are favored for certain amphibians (McMonigle 2013). This tropical South-American isopod survives in indoor conditions, and its establishment is also plausible under milder climates.

## Ecosystem services and disservices of terrestrial isopods in the urban landscape

### Regulating ecosystem services

Isopods are macro-decomposers feeding primarily on plant detritus but complementing it with other nitrogen rich resources, such as roots and tubers, vegetables, occasional green leaves, dead animal tissue and animal droppings. Together with other soil invertebrates, they accelerate plant matter decomposition and promote microbial access to organic carbon, thereby affecting nutrient turnover and soil formation. In urban parks and remnant forests they function similarly to wildland ecosystems. However, in residential areas they may be viewed differently. Some species, such as *Porcellionidespruinosus*, *Porcellionidessexfasciatus* (Budde-Lund, 1885), and *Armadillidiumvulgare*, are common inhabitants of compost heaps and manure piles (Sousa et al. 2000, Wijnhoven 2000, Achouri et al. 2008), and considered to be beneficial organisms. Tropical species can also be found in compost; an example is *Venezilloparvus* (Budde-Lund, 1885) (*V.evergladensis* in reference) in Ft. Lauderdale, USA (Johnson 1985). At the same time, because isopods occur in cellars, garages, and occasionally wander into houses, many residents view them as nuisance. In the US, pest control companies include woodlice in their list of household pests and recommend their extermination.

Urban agriculture is a fast growing phenomenon worldwide with over 800 million people practicing some form of farming or animal husbandry (FAO 2017). Small community gardens, farms spanning over several blocks, rooftop farms, and other forms of farming provide access to better quality food for inner city residents, build social cohesion in the neighborhood, and utilize abandoned land. Urban farms use large amounts of organic mulch and produce green refuse for composting, both of which are resources for terrestrial isopods, leading to them becoming permanent features in community and residential gardens. As detritivores, the commonly held view was that they cause only minor damage in vegetable and other crops (Paoletti and Hassall 1999). However, a growing number of studies indicate that under special circumstances isopod populations can grow out of control which may lead to attacking crops (Plate and Frömming 1953, Byers and Anderson 1985, Honek et al. 2009). Souty-Grosset and Faberi (in press) report that several species, for instance *Armadillidiumvulgare* and *Australiodillobifrons* (Budde-Lund, 1885) are now considered emerging pests causing significant economic damage in cereal, soybean, canola and other fields. In greenhouses isopod populations can grow exponentially especially if fresh compost is present. The woodlice then shift to and damage the organically grown vegetables as alternative food source (Messelink and Bloemhard 2007). It remains to be seen if in urban farms isopod populations reach the pest status and if their populations need to be controlled.

Isopods are food source for many invertebrate and vertebrate predators. Ground feeding birds, such as the European blackbird (*Turdusmerula* Linnaeus, 1758) especially benefit from the abundance of soil macrofauna, such as earthworms, insect larvae, isopods, and other invertebrates (e.g., Török and Ludwig 1988). In Nacogdoches, Texas, USA, isopods were one of the three major prey items in the diet of the Mediterranean house gecko, *Hemidactylusturcicus* (Linnaeus, 1758) (Saenz 1996). Even more interesting is the behavior of the ant *Leptogenyspropefalcigera* Roger, in the urban areas of Sao Vicente, Brazil. Colonies nest in building wall cracks and fissures, and feed exclusively on oniscid isopods, rejecting other invertebrates and honey as food source (Freitas 1995).

### Cultural ecosystem services

Neighborhood parks, schoolyards, university campuses community gardens and other green spaces serve as ‘living classrooms’ for children and adults. In the shrinking cities of US, low income inner city residents may not have the means to ‘venture out to nature’, they experience plant and animal life through green spaces near their homes. Urban soil biodiversity can be surprisingly high in cities, providing opportunity to demonstrate the variety of life forms, to talk about their functions, and to connect soil health and human health. Isopods are part of this conversation, because most people played with roly-polies as children, and because they are easy to observe, culture and experiment with. Citizen scientists can be actively involved in isopod surveys, add new records of species and localities, or make observations on the life cycle of their local populations, while they themselves learn about global change or conservation issues. For instance, the British Myriapod & Isopod Group (http://www.bmig.org.uk/) trains volunteers to identify and record centipedes, millipedes, pauropods, symphylans, woodlice and waterlice. The objective of the Spinicornis Project (https://www.spinicornis.be) is to build an ecological atlas of Belgian terrestrial isopods. To engage volunteers, the project organizes collecting excursions, provides simple identification key, and guidelines on how to take high quality photographs on invertebrates. Another example of citizen involvement is the Bioblitz Program, overseen by the National Geographic Society (https://www.nationalgeographic.org/projects/bioblitz/), also local environmental organizations, and agencies. Bioblitz events record local biodiversity in a short period of time, usually 24–48 hours, with the active participation of the public. In 2016, as part of the celebration of the USA National Park Service, a large scale Bioblitz was organized in Washington DC. To keep citizens engaged the group organizes regular field meetings, publishes a newsletter and bulletin, and maintains a webpage with distribution maps, images, and helpful comments for identification. Involving citizen scientists to collect scientific data is not without challenges, but utilizing this valuable resource can be very rewarding (Cohn 2008, Bonney et al. 2009).

## Research needs on urban isopod ecology and evolution

### Greater geographical coverage

Research questions in this area

What is the fraction of regional species pool persisting in urban/suburban areas?
Does urban isopod species richness exhibit a latitudinal gradient, and if so, how does it compare to trends in natural habitats?
What is the rate of species turnover of urban isopod fauna as a function of distance?

Huge gaps exist in regional and global scale distribution of isopods not only in cities but on the regional species pool, as well (Figure 2). Most European cities are old, with hundreds or even thousands years of history and major disturbances such as land use change, migration, industrialization and wars. Interpreting fauna data today is challenging in light of such long term changes. However, currently most urban land conversion takes place in the developing world, especially in coastal areas, tropical dry and moist forests, deserts, and tropical grasslands (Elmquist et al 2013). Many of these areas are biodiversity hotspots, thus the proper assessment of local extinction and colonization of species including that of terrestrial isopods in these areas is an urgent necessity.

Extending geographical scale results in examination of a broader range of climatic conditions, biomes and soil types, different cultures, economies, and human perception and value systems. Only a sufficiently large dataset enables us to examine large scale biogeographical patterns. Sfenthourakis and Hornung (2018) have shown that in Europe a latitudinal gradient exists with decreasing species numbers to the North. Moreover, biogeographical and ecological species characters shift with latitude. Endemic species, dominating in the Mediterranean, gradually disappear at higher latitudes and are replaced by habitat generalist species. Testing general hypotheses such as Biotic Homogenization in urban settings also requires large scale datasets. We do not know whether different zoogeographical/climatic regions have specific subsets of homogenizing species, or the same 8–10 species (Table 1) dominate everywhere. Preliminary surveys indicate that in the Mediterranean region *Agabiformiuslentus* (Budde-Lund, 1885) regularly occur in cities (Vilisics et al. 2012). Combining distribution records with genetic data can reveal origin, dispersal patterns, and timing of introduction of non-native species worldwide.

### City and neighborhood scale surveys

Research questions in this area

What is the relationship between city size, age, area of green spaces, and other landscape features in isopod diversity?
What is the human perception of soil macroarthropods in general, and specifically on isopods in different regions and cultures?

To answer the questions in this and the previous section, we need reliable data on isopod species richness and composition at city scale. Most studies reviewed here targeted a particular set of urban habitats, such as forest fragments, gardens or urban parks, and their species lists are likely incomplete. Many species are undocumented because they are rare, i.e. their abundance is low, and/or present only in a few, specialized landscape patches, such nearby historical ruins, greenhouses, or sewer drains (Kontschán 2004, Vilisics 2007). Assessments of epigeic arthropod communities usually rely on pitfall trap material. Smaller isopod species rarely fall into those, and thus will be underestimated. It is very important to complement pitfall trap sampling with other methods such as leaf litter sifting and traditional hand collecting that targets unusual habitats. Recently in Manaus, Brazil, Ogawa (2008) found isopods in the nest of the urban pigeon, *Columbalivia* Gmelin, 1789. More sampling points increases the coverage on the landscape, and even though unless standardized, hand sampling usually provides only presence-absence data, the true species richness of the city and suburban areas will be better captured.

### Diversity and stability of isopod assemblages

Research questions in this area

What is the relative contribution of local environmental factors (soil type, vegetation, microclimate) and management (irrigation, amendments, pesticide use, litter collection) in determining composition and abundance of isopod assemblages?
What is the role of corridors in dispersal and exchange of individuals among local populations? Do isopods use grey infrastructure (buildings, underground conduits) to disperse?
What are the key landscape properties ensuring long term persistence of isopod populations, and how do these vary with climatic conditions?

The urbanized landscape is highly fragmented leading to isolation of communities. Species richness of these isolated patches can be highly variable from one species to ten or more. Priority effect can play a major role in which species get established. Isopods are being moved around with soil, plants, mulch and other landscaping materials. Consequently, species presence in a local patch might be determined by ‘who gets there first’. Soil invertebrate surveys in cities are often campaign-like, such as the above mentioned Bioblitz efforts, which provide a snapshot of the local community. We know essentially nothing about the persistence of these populations. Long term field monitoring is needed to reveal how patterns of alpha and beta diversity change over time and how stable urban isopod communities are. For arthropods in general, local landscape features have been shown to drive local diversity, although the specific drivers are taxon dependent (Smith et al. 2006a, Philpott et al. 2014). A complex habitat creates more diverse resources and microhabitat conditions allowing coexistence of more species (Byrne 2007, Ossola et al. 2016). Research is needed to explore the role of habitat structure and the defining components of the local landscape specifically on terrestrial isopod assemblages. Experimental data show that the probability of success to increase biodiversity varies by the type of design changes as well as by the targeted taxa (Gaston et al. 2005). This knowledge is also important for practitioners when making decisions about urban green spaces for promoting local diversity, protecting rare species or enhancing particular ecosystem services (Snyder and Hendrix 2008, Szlavecz et al. 2018).

### Function of isopods in novel ecosystems

Research questions in this area

Given their often high abundances, what is the role of terrestrial isopods in nutrient turnover, especially in regions lacking earthworms?
Under what circumstances can isopod become pests in urban gardens, local crop fields, and greenhouses?

The urban landscape is dominated by highly manipulated, engineered, and built components designed by humans to serve a given purpose. Soils are often engineered to support buildings and roads, to plant street trees, to establish green roofs, or to intercept and retain water. A particular example of the latter is natural water treatment systems such as rain gardens and bioswales. These novel ecosystems are readily colonized by soil fauna, including isopods (Ayers 2009, Mehring and Levin 2015) but we do not know if their presence and activity enhance or reduce such functions as nutrient release and retention, organic matter processing, infiltration, contaminant and pathogen removal, and plant growth. Woodlice might also have an important role in distributing mycorrhizal propagules (Rabatin and Stinner 1988, 1989). The role of soil fauna in the efficiency in these engineered systems needs to be quantified, and, combined with ecological theory, living organisms should be included in the design process (Levin and Mehring 2015).

In general, the urban soil food web is highly altered, because the resource base is altered. On the one hand, leaf litter, a major food source for terrestrial isopods, is removed from lawns and impervious surfaces. On the other hand compost, mulch, manure and other landscaping material create a concentrated abundant resource supply. How does this affect spatio-temporal abundance of terrestrial isopods? A related issue is the relative role of urban isopods in decomposition and nutrient turnover. Grass/lawn is the dominating land cover type in temperate urban/suburban areas. Pieper and Weigmann (2008) have shown that the presence of *Porcellioscaber* and two Collembola species resulted in faster decomposition of grass litter, greater losses of most cations, but higher retention of organic carbon in urban soil, but such mesocosm studies need to be extended to long-term field investigations.

### Adaptation and evolution in the urban environment

Research questions in this area

What environmental factors in an urban environment can act as selective forces?
What are the key biological traits contributing to the success of some urban isopod species?
Are life history characteristics of urban and corresponding rural populations of isopods different?
Are urban and rural isopod populations genetically different?

Humans are major drivers of both adaptive and non-adaptive evolutionary change. Urban evolution is an emerging field focusing on individual and population responses to urban selective forces, and the underlying micro-evolutionary changes. So far research has been heavily biased towards vertebrates, and plants (McDonnell and Hahs 2015), with invertebrate studies lagging behind (Johnson and Munshi-South 2017). Isopods are excellent model organisms to study mechanisms of evolutionary change because of their ubiquitous occurrence, somewhat limited dispersal ability, and demonstrated responses to known urban stressors, such as pollution and the altered urban climate. Moreover, their reproductive cycle is strongly driven by light regimes (e.g., McQueen and Steel 1980, Warburg et al. 1984, Juchault et al. 1985), and they have been shown to alter their behavior to increased vibration, a much less studied urban effect (Houghtaling and Kight 2006, Cividini and Montesanto 2018a, 2018b). Successful establishment and range expansion of terrestrial isopods depends on their reproductive strategies, phenotypic plasticity, and omnivore feeding habits (Rushton and Hassall 1983, Hornung and Warburg 1998, Hornung 2011). Species with wider tolerance limits, higher reproductive output, and with the ability of sperm storage (Suzuki and Ziegler 2005) or being parthenogenetic have a better chance for colonization and geographical range expansion. To separate true evolutionary response from phenotypic plasticity, which is commonly exhibited in isopods, molecular studies are needed.

## Conclusions

Terrestrial isopods are ubiquitous members of the epigeic soil fauna in cities. They are well established in the built environment and in all types of urban green spaces including remnant habitat patches, parks, residential yards, vacant lots, and greenhouses. Urban isopod assemblages are a mixture of a few cosmopolitan species that thrive in human dominated landscapes, a subset of the native fauna, and more recently introduced species. The urbanized landscape is highly fragmented, leading to isolation of communities. Alpha diversity of these isolated patches varies, but comparable to species richness in more natural areas. At the same time, species turnover among the habitat patches can be high resulting in high species richness at city scale. These observations are highly biased, because the overwhelming majority of data were collected in the temperate zone. Globally, distribution of isopods is limited at higher latitudes due to cold temperatures. However, current warming trends coupled with urbanization that provides refuges from extreme conditions, are pushing these boundaries northward.

Synanthropic species thrive in the city, often dominating the detritivore macrofauna, but special habitats can be refuges for native species, as well. The urban setting provides an excellent opportunity to study the dynamics of spatially isolated communities, the underlying mechanisms of local extinction, colonization, and dispersal, and the role of human perception, disturbance and management plays in these processes. As abundant, often dominant detritivores, their role in decomposition and nutrient release needs to be studied especially in engineered ecosystems. Future research should also include eco-evolutionary changes, preferably in the rapidly urbanizing regions in the world.

## Supplementary materials

Supplementary material 1 List of cities with terrestrial isopod records Data type: occurrence Explanation note: Publications older than 70 years are not included. Localities listed here are mapped on Fig. 2 in the text. Source of geographical coordinates and population data: https://latitude.to This is an open access article distributed under the terms of the Creative Commons Attribution License (CC BY 4.0), which permits unrestricted use, distribution, and reproduction in any medium, provided the original author and source are credited.Katalin Szlavecz, Ferenc Vilisics, Zsolt Tóth, Elisabeth Hornung

Supplementary material 2 List of terrestrial isopod species records in urban areas Data type: occurrence Explanation note: Species name have been cross-checked with the world catalog by Schmalfuss (2003). Synonyms have been changed to valid species names. In some cases the species was not listed in the catalog. Notes regarding uncertainties are also from Schmalfuss (2003). This is an open access article distributed under the terms of the Creative Commons Attribution License (CC BY 4.0), which permits unrestricted use, distribution, and reproduction in any medium, provided the original author and source are credited.Katalin Szlavecz, Ferenc Vilisics, Zsolt Tóth, Elisabeth Hornung
