# Supplementary material for: Terrestrial isopods in urban environments: an overview
Source: Zookeys. 2018 Dec 3;(801):97–126. doi: 10.3897/zookeys.801.29580 (PMC6288257; doi:10.3897/zookeys.801.29580)
Supplement: Supplementary material 1 — List of cities with terrestrial isopod records [file zookeys-801-097-s001.docx]

Supplementary Table 1. List of cities with terrestrial isopod records. Publications older than 70 years are not included. Localities listed here are mapped on Fig. 2 in the text. Source of geographical coordinates and population data: [https://latitude.to](https://latitude.to/)

| **City** | **Country** | **Latitude** | **Longitude** | **Population** | **References** |
| --- | --- | --- | --- | --- | --- |
| Aberdeen | USA | 45.46 | -98.49 | 26,091 | Wright 1997 |
| Åland | Finland | 19.93 | 60.10 | 26,711 | Vilisics and Terhiuvo 2009 |
| Ann Arbor | USA | 42.22 | -83.75 | 113,934 | Hatchett 1947 |
| Amsterdam | Netherland | 4.90 | 52.38 | 832,563 | M. Berg, pers. comm. |
| Auckland | New Zealand | 174.76 | -36.85 | 417,910 | Scott 1984 |
| Baltimore | USA | -76.61 | 39.30 | 620,961 | Hornung and Szlavecz 2003,  Hornung et al. 2015, Szlavecz unpubl. |
| Beiuş | Romania | 22.35 | 46.67 | 10,721 | Bodin et al. 2013 |
| Berlin | Germany | 13.41 | 52.52 | 3,426,354 | Fritsche 1936 |
| Bonn-Bad Godesberg | Germany | 7.15 | 50.683 | 73,172 | Schulte et al. 1989 |
| Bucharest | Romania | 26.11 | 44.43 | 1,877,155 | Giurginca 2006 |
| Budapest | Hungary | 19.04 | 47.50 | 1,741,041 | Korsós et al. 2002,  Vilisics and Hornung, 2009,  Hornung et al. 2007 |
| Debrecen | Hungary | 21.63 | 47.53 | 204,124 | Magura et al. 2008 |
| Gaborone | Botswana | 25.91 | -24.65 | 231,626 | Dangerfield and Telford 1995 |
| Gdynia | Poland | 18.53 | 54.52 | 253,730 | M. Rybak, pers. comm. |
| Göttingen | Germany | 9.93 | 51.53 | 122,149 | Schaefer 1982 |
| Haifa | Israel | 34.99 | 32.82 | 277,082 | Warburg and Hornung 1999 |
| Harare | Zimbabwe | 31.05 | -17.83 | 1,606,000 | Dangerfield and Telford 1995 |
| Helsinki | Finland | 24.95 | 60.19 | 626,305 | Vilisics and Terhiuvo 2009 |
| Kaunas | Lithuania | 23.90 | 54.90 | 374,643 | Šatkauskienė et al. 2015 |
| Kiel | Germany | 10.13 | 54.32 | 240,832 | Tischler 1980 |
| Krakow | Poland | 19.94 | 50.06 | 755,050 | A. Antoł and M. Bonk, pers. comm. |
| Lapua | Finland | 23.01 | 62.97 | 14,091 | Vilisics and Terhiuvo 2009 |
| Leipzig | Germany | 12.37 | 51.34 | 504,971 | Beyer 1957, Arndt and Mattern 1996 |
| London | United Kingdom | -0.13 | 51.51 | 7,556,900 | Smith et al. 2006 |
| Luzern | Switzerland | 8.30 | 47.05 | 57,066 | Vilisics et al. 2012 |
| Lugano | Switzerland | 8.86 | 46.00 | 26,365 | Vilisics et al. 2012 |
| Mariánské Lázně | Czech Republic | 12.70 | 49.96 | 14,277 | L. Dvorak, pers. comm. |
| Medieşu Aurit | Romania | 23.15 | 47.78 | 7,122 | Ferenţi and Covaciu-Markov 2013 |
| Melbourne | Australia | 144.96 | -37.81 | 4,246,375 | Norton et al. 2014 |
| Moscow | Russia | 37.62 | 55.75 | 10,381,222 | Zalesskaya and Rybalov 1982,  Gongalsky and Kuznetsova 2010,  Gongalsky et al. 2013 |
| Munakata City | Japan | 130.53 | 33.8 | 96,728 | Nasu et al. in press |
| Olomouc | Czech Republic | 17.25 | 49.60 | 101,268 | Riedel et al. 2009 |
| Palmerston North | New Zealand | -40.36 | 175.61 | 87,300 | Parker and Minor 2015 |
| Pâncota | Romania | 21.70 | 46.33 | 7,133 | Laza et al. 2017 |
| Pécs | Hungary | 18.25 | 46.08 | 148,021 | Farkas and Vilisics 2006 |
| Pisa | Italy | 10.40 | 43.71 | 77,007 | Montesanto 2015 |
| Porto Allegre Rio Grande do Sul | Brazil | -51.23 | -30.03 | 1,372,741 | P.B. Araujo, pers. comm. |
| Porvoo | Finland | 25.67 | 60.39 | 47,192 | Vilisics and Terhiuvo 2009 |
| Riga | Latvia | 24.11 | 56.95 | 742,572 | Spungis, 2008 |
| Salonta | Romania | 21.65 | 46.80 | 17,561 | Ferenţi et al. 2015 |
| Sorø | Denmark | 11.56 | 55.44 | 7,754 | Vilisics et al. 2007 |
| Stalowa Wola | Poland | 22.05 | 50.57 | 64,353 | M. Rybak, pers. comm. |
| Ștei | Romania | 22.45 | 46.53 | 6,529 | Herle et al. 2016 |
| Szczecin | Poland | 14.55 | 53.43 | 407,811 | L. Sługocki, pers. comm. |
| Tokyo | Japan | 139.69 | 35.69 | 8,336,599 | Nonomura 2005a, 2005b |
| Toyama City | Japan | 137.22 | 36.70 | 325,532 | Nunomura 1980 |
| Vienna | Austria | 16.37 | 48.21 | 1,691,468 | Kühnelt 1989 |
| Vilnius | Lithuania | 25.28 | 54.69 | 542,366 | Kuznetsova and Gongalsky 2012 |
| Warsaw | Poland | 21.02 | 52.24 | 1,702,139 | Jedryczkowski 1981 |
| Zurich | Switzerland | 8.54 | 47.38 | 341,730 | Vilisics et al. 2012 |

**References to Supplementary table 1.** Publications, not referenced the main text, are listed below.

Beyer R (1958) Ökologische und brutbiologische Untersuchungen an Landisopoden der Umgebung von Leipzig. Wissenschaftliche Zeitschrift der Karl-Marx-Universität Mathematisch-naturwissenschaftliche Reihe 7: 291–308.

Bodin AA, Ferenţi S, Ianc R, Covaciu-Marcov SD (2013) Some data upon the herpetofauna and terrestrial isopods from Beiuş town, Romania. South-Western Journal of Horticulture, Biology and Envorinment 4 (2): 137–149.

Dangerfield JM, Telford SR (1995) Tactics of reproduction and reproductive allocation in four species of woodlice from southern Africa. Journal of Tropical Ecology 11 (4): 641–649.

Farkas S, Vilisics F (2006). A Mecsek szárazföldi ászkarák együttesei (Isopoda: Oniscidea). (The terrestrial isopod fauna of the Mecsek Mts.) Folia comloensis, 15: 25–34. (in Hungarian)

Ferenţi S, Covaciu-Marcov SD (2013) Travelling Isopods: *Oniscus asellus* (Crustacea, Isopoda) in an Anthropogenic Habitat from North-Western Romania. Entomologica Romanica 18: 11–13.

Ferenţi S, Lucaciu M, Mihuţ A (2015). Terrestrial isopods from Salonta town, Western Romania. South-Western Journal of Horticulture. Biology and Environment, 6 (1): 21–31.

Fritsche H (1936) Beiträge zur Oekologie der Land-Isopoden Groß-Berlins. Märkische Tierwelt 2(2): 65-117.

Gongalsky KB, Kuznetsova DM (2010) Additions to the woodlice fauna (Isopoda: Oniscidea) of Moscow region. Byulleten' Moskovskogo Obshchestva Ispytatelei Prirody Otdel Biologicheskii 115 (3): 46–47.

Gongalsky KB, Kuznetsova DM, Filimonova ZhV, Shakhab SV (2013) Distribution and ecology of the invasive species of woodlice Hyloniscus riparius (C. Koch, 1838) (Isopoda, Oniscidea, Trichoniscidae) in Russia. Russian Journal of Biological Invasions. 6 (1): 2–7. doi:10.1134/S2075111713020045

Herle AI, Covaciu-Marcov S-D, Ferenţi S (2016) Past industry vs. nature: which one influences

more the terrestrial isopod assemblages from a town in western Romania? Oltenia. Studii și comunicări.

Științele Naturii. Muzeul Olteniei Craiova 32(1):55–60.

Laza D, Popovici PV, Bodog DE, Molnár K, Ferenţi S (2017) Terrestrial isopods in a small town in western Romania (Pâncota, Arad county): Witnesses of the past human impact of the region? Muzeul Olteniei Craiova. Oltenia. Studii şi comunicări. Ştiinţele Naturii 33 (2)

Nasu T, Kitagawa K, Karasawa S (in press): Species compositions of terrestrial isopods in public parks of a commuter town in Japan, ZooKeys

Nunomura N (1980) Some terrestrial isopod crustaceans from Toyama City, Middle Japan. Bulletin of the Toyama Science Museum 2: 13–21.

Nunomura, N (2005a) Terrestrial isopod and amphipod crustaceans from the Akasaka Imperial

Gardens, Tokyo, Japan. Memoirs of the National Science Museum (Tokyo) 39: 491-494 (in Japanese with English summary)

Nunomura, N (2005b) Terrestrial isopod crustaceans from the Tokiwamatsu Imperial Villa, Tokyo, Japan. Memoirs of the National Science Museum (Tokyo) 39: 495-498 (in Japanese with English summary)

Parker AN, Minor MA (2015) Native and adventive detritivores (Diplopoda, Isopoda and Amphipoda) in a modified landscape: influence of forest type and edge. New Zealand Journal of Ecology, 39 (2): 323–331.

Šatkauskienė I, Hornung E, Lelešius E, Kvašnauskaitė K, Ašmantas Š (2016) Preliminary study on the terrestrial isopods of Kaunas city (Lithuania). Zoology and Ecology 26 (1): 22–27.

Scott RR (1984) The isopod fauna of some Auckland reserves and areas of native vegetation. Tane 30: 227.

Spuņģis V (2008) Fauna, distribution, habitat preference and abundance of woodlice (Oniscidea) in Latvia. Latvijas entomologs 45: 25–37.

Warburg MR, Hornung E (1999) Diversity of terrestrial isopod species along a transect through northern Israel. Biodiversity and Conservation 8: 1469–1478

Zalesskaya NT, Rybalov LV. (1982) Fauna of woodlice of Moscow Region (Crustacea, Isopoda, Oniscidea). In: Gilyarov MS (Ed) Soil invertebrates of Moscow Region. Nauka Publishers, Moscow, pp. 171–179. [in Russian]
