# Supplementary material for: Terrestrial isopods in urban environments: an overview
Source: Zookeys. 2018 Dec 3;(801):97–126. doi: 10.3897/zookeys.801.29580 (PMC6288257; doi:10.3897/zookeys.801.29580)
Supplement: Supplementary material 2 — List of terrestrial isopod species records in urban areas [file zookeys-801-097-s002.docx]

Supplementary Table 2. List of terrestrial isopod records in urban areas. Species name have been cross-checked with the world catalog by Schmalfuss (2003). Synonyms have been changed to valid species names. In some cases the species was not listed in the catalog. Notes regarding uncertainties are also from Schmalfuss (2003).

| **Family** | **Species** |
| --- | --- |
| Agnaridae | *Agnara pannuosa* (Nunomura, 1987) |
|  | *Lucasioides tokyoensis* Nunomura, 2000 |
|  | *Mongoloniscus katakurai* (Nunomura, 1987) |
|  | *Mongoloniscus koreanus* Verhoeff, 1930 |
|  | *Mongoloniscus maculatus* (Iwamoto, 1943) |
|  | *Orthometopon planum* (Budde-Lund, 1885) |
|  | *Protracheoniscus major* (Dollfus, 1903) |
|  | *Protracheoniscus politus* (C. Koch, 1841) |
|  |  |
| Armadillidae | *Armadillo albus* Schmalfuss, 1996 |
|  | *Armadillo carmelensis* Schmalfuss, 1996 |
|  | *Armadillo officinalis* Duméril, 1816 |
|  | *Venezillo obscurus* (Budde-Lund, 1885) |
|  |  |
| Armadillidiidae | *Armadillidium album* Dollfuss, 1887 |
|  | *Armadillidium depressum* Brandt, 1833 |
|  | *Armadillidium marmoratum* Strouhal, 1929 |
|  | *Armadillidium nasatum* Budde-Lund, 1885 |
|  | *Armadillidium opacum* (C. Koch, 1841) |
|  | *Armadillidium pictum* Brandt, 1883 |
|  | *Armadillidium pulchellum* (Zenker, 1798) |
|  | *Armadillidium versicolor* Stein, 1859 |
|  | *Armadillidium vulgare* (Latreille, 1804) |
|  | *Armadillidium zenckeri* Brandt, 1833 |
|  | *Bethalus pretoriensis* (Dollfus, 1895) |
|  | *Eluma caelatum* (Miers, 1877) |
|  | *Schizidium tiberianum* Verhoeff, 1923 |
|  |  |
| Balloniscidae | *Balloniscus glaber* Araujo and Zardo, 1996 |
|  | *Balloniscus sellowii* (Brandt, 1833) |
|  |  |
| Bathytropidae | *Bathytropa wahrmani* Strouhal, 1968 |
|  | *Neotroponiscus daguerrii* Giambiagi de Calabrese 1939 |
|  |  |
| Cylisticidae | *Cylisticus convexus* (De Geer, 1778) |
|  | *Cylisticus transsilvanicus* Verhoeff, 1908 |
|  |  |
| Dubioniscidae | *Novamundoniscus gracilis* Lopes and Araujo, 2003 |
|  |  |
| Ligiidae | *Ligia oceanica* (Linnaeus, 1767) |
|  | *Ligidium germanicum* Verhoeff, 1901 |
|  | *Ligidium hypnorum* (Cuvier, 1792) |
|  | *Ligidium japonicum* Verhoeff, 1918 |
|  | *Ligidium koreanum* Flasarová, 1972 |
|  |  |
| Oniscidae | *Oniscus asellus* Linnaeus, 1758 |
|  | *Sardoniscus verhoeffi* (Ferrara and Taiti, 1978) |
|  |  |
| Plathyarthridae | *Platyarthrus attanassovi* Verhoeff, 1936 |
|  | *Platyarthrus aiasensis* Legrand, 1954 |
|  | *Platyarthrus hoffmannseggii* Brandt,1883 |
|  | *Platyarthrus schoblii* Budde-Lund, 1885 |
|  | *Trichorhina argentina* Vandel, 1963 |
|  | *Trichorhina tomentosa* (Budde-Lund, 1893) |
|  |  |
| Philosciidae | *Anchiphiloscia balssi* (Verhoeff, 1928) |
|  | *Aphiloscia vilis* (Budde-Lund, 1885) |
|  | *Atlantoscia floridana* (Van Name, 1940) |
|  | *Atlantoscia inflata* Campos-Filho and Araujo, 2015 |
|  | *Atlantoscia petronioi* Campos-Filho, Contreira and Lopes-Leitzke, 2012 |
|  | *Burmoniscus kathmandius* (Schmalfuss, 1983) |
|  | *Burmoniscus meeusei* (Holthuis, 1947) |
|  | *Chaetophiloscia cellaria* (Dollfus, 1884) |
|  | *Chaetophiloscia elongata* (Dollfus, 1884) |
|  | *Chaetophiloscia elongata* (Dollfus, 1884) |
|  | *Chaetophiloscia lagoi* (Arcangeli, 1934) |
|  | *Chaetophiloscia sicula* Verhoeff, 1908 |
|  | *Chaetophiloscia warburgi* Schmalfuss, 1991 |
|  | *Lepidoniscus minutus* (C. Koch, 1838) |
|  | *Paraphiloscia pubescens* (Dana, 1853)† |
|  | *Philoscia affinis* Verhoeff, 1908 |
|  | *Philoscia muscorum* (Scopoli, 1763) |
|  | *Pseudophiloscia brevicornis* Budde-Lund, 1912) |
|  |  |
| Porcellionidae | *Agabiformius lentus* (Budde-Lund, 1885) |
|  | *Agabiformius orientalis* (Dollfus, 1905) |
|  | *Leptrotrichus naupliensis* (Verhoeff, 1901) |
|  | *Leptotrichus panzerii* (Audouin, 1826) |
|  | *Porcellio dilatatus* Brandt, 1833 |
|  | *Porcellio ficulneus* Budde-Lund, 1885 |
|  | *Porcellio laevis* (Latreille, 1804) |
|  | *Porcellio scaber* Latreille, 1804 |
|  | *Porcellio spinicornis* Say, 1818 |
|  | *Porcellionides myrmecophilus* (Stein, 1859) |
|  | *Porcellionides pruinosus* (Brandt, 1833) |
|  | *Porcellionides trifasciatus* (Dollfus, 1892) |
|  | *Proporcellio quadriseriatus* Vehoeff, 1917 |
|  |  |
| Styloniscidae | *Cordioniscus stebbingi* (Patience, 1907) |
|  | *Styloniscus otakensis* (Chilton, 1901) |
|  | *Styloniscus phormianus* (Chilton, 1901) |
|  | *Styloniscus spinosus*(Patience, 1907) |
|  | *Styloniscus thomsoni* (Chilton, 1885) |
|  |  |
| Trachelipodidae | *Nagurus carinatus* (Dollfus, 1905) |
|  | *Porcellium collicola* (Verhoeff, 1907) |
|  | *Porcellium conspersum* (C. Koch, 1841) |
|  | *Trachelipus arcuatus* (Budde-Lund, 1885) |
|  | *Trachelipus nodulosus* (C. Koch, 1838) |
|  | *Trachelipus rathkii* (Brandt, 1833) |
|  | *Trachelipus ratzeburgii* (Brandt, 1833) |
|  | *Trachelipus razzautii* (Arcangeli, 1913) |
|  | *Trachelipus rhinoceros* (Budde-Lund, 1885) |
|  |  |
| Trichoniscidae | *Androniscus dentiger* Verhoef, 1908 |
|  | *Androniscus roseus* (C. Koch,1838) |
|  | *Buddellundiella cataractae* Verhoeff, 1930 |
|  | *Haplophthalmus danicus* Budde-Lund, 1880 |
|  | *Haplophthalmus mengii* (Zaddach, 1844) |
|  | *Haplophthalmus montivagus* Verhoeff, 1941 |
|  | *Hyloniscus riparius* (C. Koch, 1838) |
|  | *Hyloniscus transsylvanicus* (Verhoeff, 1901) |
|  | *Hyloniscus vividus* (C. Koch, 1841) |
|  | *Metatrichoniscoides leydigi* (Weber, 1880) |
|  | *Miktoniscus linearis* (Patience, 1908) |
|  | *Miktoniscus medcofi* Van Name, 1940 |
|  | *Miktoniscus patiencei* Vandel, 1946 |
|  | *Trichoniscus alemannicus* Verhoeff, 1917 |
|  | *Trichoniscus noricus* Verhoeff, 1917 |
|  | *Trichoniscus pusillus* Brandt, 1833 |
|  | *Trichoniscus pygmaeus* Sars, 1899 |
|  | *Trichoniscoides albidus* (Budde-Lund, 1880) |
|  | *Trichoniscoides helveticus* (Carl, 1908) |
|  | *Trichoniscoides sarsi* Patience, 1908 |
|  |  |
| Crinocheta, ‡ | *Exalloniscus cortii* Arcangeli, 1927 |

† Dubious name

‡ Family designation uncertain
